# Home range size of Tengmalm’s owl during breeding in Central Europe is determined by prey abundance

**DOI:** 10.1371/journal.pone.0177314

**Published:** 2017-05-18

**Authors:** Marek Kouba, Luděk Bartoš, Václav Tomášek, Alena Popelková, Karel Šťastný, Markéta Zárybnická

**Affiliations:** 1 Department of Animal Science and Ethology, Faculty of Agrobiology, Food and Natural Resources, Czech University of Life Sciences Prague, Prague, Czech Republic; 2 Department of Ecology, Faculty of Environmental Sciences, Czech University of Life Sciences Prague, Prague, Czech Republic; 3 Department of Ethology, Institute of Animal Science, Prague, Czech Republic; 4 Nature Conservation Agency of the Czech Republic, Prague, Czech Republic; 5 Department of Applied Geoinformatics and Spatial Planning, Faculty of Environmental Sciences, Czech University of Life Sciences Prague, Prague, Czech Republic; University of Lleida, SPAIN

## Abstract

Animal home ranges typically characterized by their size, shape and a given time interval can be affected by many different biotic and abiotic factors. However, despite the fact that many studies have addressed home ranges, our knowledge of the factors influencing the size of area occupied by different animals is, in many cases, still quite poor, especially among raptors. Using radio-telemetry (VHF; 2.1 g tail-mounted tags) we studied movements of 20 Tengmalm’s owl (*Aegolius funereus*) males during the breeding season in a mountain area of Central Europe (the Czech Republic, the Ore Mountains: 50° 40’ N, 13° 35’ E) between years 2006–2010, determined their average hunting home range size and explored what factors affected the size of home range utilised. The mean breeding home range size calculated according to 95% fixed kernel density estimator was 190.7 ± 65.7 ha (± SD) with a median value of 187.1 ha. Home range size was affected by prey abundance, presence or absence of polygyny, the number of fledglings, and weather conditions. Home range size increased with decreasing prey abundance. Polygynously mated males had overall larger home range than those mated monogamously, and individuals with more fledged young possessed larger home range compared to those with fewer raised fledglings. Finally, we found that home ranges recorded during harsh weather (nights with strong wind speed and/or heavy rain) were smaller in size than those registered during better weather. Overall, the results provide novel insights into what factors may influence home range size and emphasize the prey abundance as a key factor for breeding dynamics in Tengmalm’s owl.

## Introduction

As early as Darwin [1] it was noted that the primary characteristic of animal movement is that most animals use the same areas repeatedly over time. Movements of this type in fairly well-defined areas within which animals perform their daily activities are often defined using the home range concept [2]. The first definition of home range (hereafter HR) was provided by Burt [3] as: “Area traversed by the individual in its normal activities of food gathering, mating, and caring for young. Occasional sallies outside the area, perhaps exploratory in nature, should not be considered part of the home range.” Although this basic construct is retained within the concept of home range to this day, it has usually been refined to include clear definition of the timeframe involved in a given home range analysis (daily, seasonal, annual, life-time etc.) and in more formal statistical analysis of HR size (e.g., [4–6]). The HR is characterized typically with descriptors of its size, shape and structure [7], and must be defined for a specific time interval [2,8,9].

Different biotic and abiotic factors (intrinsic and/or extrinsic) are likely to affect the size, use, and spatial configuration of individuals’ HR, and all these factors interact along a hierarchical pattern according to different spatial and temporal scale [10–12]. Using hierarchy theory [13], McLoughlin and Ferguson [10] offer review of the limiting factors that likely determine HR size at three spatial levels (among species, populations and individuals) identifying the critical factors as including (*inter alia*): body size, climate, abundance and distribution of food, social organisation, population density and risk of predation.

At the species level, positive correlations between size of HR and body mass/size were found in mammals, birds and lizards (e.g., [14–16]), with carnivores as a rule found to have larger HRs compare to herbivores (reviewed by [17]). At the population level, major determinants of HRs include climate and its effects on general habitat productivity, density, and the spatial structure of the environment such as primary productivity, seasonality, and food availability/accessibility (reviewed by [10]). At the individual level, food availability, conspecific density, and risk of predation are likely the primary determinants of HR size (reviewed by [10]). In birds and mammals an inverse relationship between HR size and food availability have been frequently found (e.g., [18–21]).

Resource (particularly food) dispersion and abundance affected, largely independently, the group size and HR area/territory size of socially living carnivores (reviewed by [22]). For instance in the European badger (*Meles meles*) the dispersion of pasture patches with high earthworm availability positively correlated with territory size, however, the group size depended on the quality of particular foraging patches within the territory [23]. In Pallas’s cat (*Otocolobus manul*) males inhabited HRs 4–5 times the size of female, smaller HRs were associated with higher coverage of preferred rocky habitats in the HR centre, whereas larger HRs were associated with higher connectivity of rocky habitats in their periphery, and HR size did not increase in response to low prey abundance or seasonality [24]. Subadult brown bear (*Ursus arctos*) males had larger ranges than females, HRs increased with increasing body size, decreased with increasing population density, but were not related to a general index of food availability and individual age [25].

In elk (*Cervus canadensis*) ranging patterns reflected complex trade-offs that affect foraging, group dynamics, movement energetics, predation avoidance and thermal regulation [26]. The HR size in red deer (*Cervus elaphus*) decreased with increasing conspecifics density, supplemental feeding intensity, average annual temperature, and males had a larger HR than females [27]. The percentage of grassland and the slope of grasslands within the HR were the main determinants of HR size in male Alpine ibex (*Capra ibex ibex*), explaining also the differences between seasons so that HR size in winter and spring was inversely correlated with the amount of snow depth while in other seasons it was linked to resource exploitation [28]. In Arctic ground squirrel (*Spermophilus parryii*) the HR size was 2–7 times smaller when individuals were food-supplemented regardless of whether large mammalian predators were present or not [29], and the eastern indigo snake (*Drymarchon couperi*) used much smaller HRs in fragmented landscapes and vice versa [30].

In birds of prey, there are many studies which have addressed home range (e.g., [31–33]), however, those which have examined also factors affecting range size are scarce and their results are often contradictory [34–40].

The Tengmalm’s owl (*Aegolius funereus*) is a small, nocturnal, cavity-nesting owl (male body mass ca. 100 g), living in coniferous forests in the boreal zone and in alpine forests further south in Eurasia [41]; it feeds mainly on small mammals [42–44]. The young stay in the nest for 27–38 days after hatching [45], and reach independence 5–9 weeks after fledging [46–49]. The great majority of prey brought to the young throughout the late nestling and post-fledging dependence period (hereafter PFDP), in this particular species, is delivered by the male [47,48,50], and during good food years polygyny may occur [43,44]. Tengmalm’s owl searches for prey by the pause-travel mode and locates it by sound [51–53].

Studies of the hunting HRs of Tengmalm’s owl males during breeding are essentially agreed on an average size of ca. 2 km^2^. Older studies which established HRs by the minimum convex polygon method [54] give the size of the hunting range as 205 ha [55], 181 ± 48 ha (mean ± SD; [56]) and 100–300 ha [57]; the study by Santangeli et al. [34] which determined the size of hunting range of males by the kernel density estimator [58] suggested a range size of 114 ± 20 ha (mean ± SE). Hakkarainen et al. [59] noticed that in the low phase of vole cycle males hunt up to 4 km from the nest, whereas in good vole years hunting trips are about one-third of that distance.

In this paper, we explore what factors determine the size of hunting HR in Tengmalm’s owl males during breeding season in different years with contrasting prey abundance.

We predicted that:

1. HRs will be larger during years with low prey abundance since during such years the males will need to hunt over larger areas to bring enough prey items to the nest [49,59].
2. Polygynous males will have larger HRs compared to males mated monogamously since the former ones have to care for spatially distant broods and usually also for more young.
3. The HR size will be affected by the number of hatchlings/fledglings raised since more numerous broods need more prey items to survive and the males will thus need to hunt on a larger area in order to feed their offspring.
4. Similarly, HR size will increase with the actual age of nestlings/fledglings since older young will need proportionally more food than younger ones, forcing the males to hunt over larger areas. Further, one could also expect the HR size will extend after fledging of offspring since the males will no longer be tied to their nest-site as the necessary core of their hunting range [49].

Finally, we also predicted that (v) the HRs will be smaller when recorded during harsh weather conditions, especially during rainy nights and/or strong wind speed; as reported by Klaus et al. [60], as little as 5 mm of rain per day caused a decrease in nest feeding visits in Tengmalm’s owl which presumably implies that hunting itself was restricted.

## Materials and methods

### Study area

The study was carried out during five breeding seasons 2006–2010 in an area close to the water reservoir Fláje in the Ore Mountains, the Czech Republic (50° 40’ N, 13° 35’ E). This area was severely damaged by air-pollution in the 1970s, with most coniferous trees above the altitude of 500 m a. s. l. dying out as a result; the study area (110 km^2^, 730–960 m a. s. l.) has been artificially replanted, with the predominant species being blue spruce (*Picea pungens*, occupying approximately 28% of the study area), Norway spruce (*Picea abies*, 26%), birch (*Betula* sp., 11%), European mountain ash (*Sorbus aucuparia*, 5%), European beech (*Fagus sylvatica*, 4%) and European larch (*Larix decidua*, 4%). Outside the forested parts the vegetation is dominated by wood reeds (*Calamagrostis villosa*) and solitary European beech [61]. To compensate for the lack of natural tree cavities, 233 wooden nestboxes lined with wood chips (with the base 25x25 cm, height 40 cm and with an entrance hole 8 cm in diameter) have been installed gradually in the area since 1999, and virtually the whole local population of Tengmalm’s owl breeds in these nestboxes.

Weather data were obtained from the closest weather stations to the study area. The average daily temperature (°C) and wind speed (m/s) were taken from the station in Nová Ves v Horách, located ca. 5 km from the study area. Daily precipitation (mm) was taken from the station in Český Jiřetín, located ca. 1.5 km from the study area.

### Field and laboratory procedures

In all study years, all nestboxes were visited at intervals of 2–3 weeks from early March to July to find nests, and thereafter, nests were checked 1–2 times per week to know the number of eggs, hatchlings and fledglings and to determine exact hatching date (± 1 day). Twenty males in total (5, 4, 4, 2 and 5 in 2006–2010, respectively) were captured during nestling phase by using mist net placed in front of the nestbox or swing-door trap placed at the entrance of the nestbox. This was done during the night when males were bringing prey items to the nest. Captured males were ringed, weighed, the length of wing was measured, and age estimated according to the method of Hörnfeldt et al. [62], before being fitted with tail-mount transmitters of type TW-4 (Biotrack Ltd., UK). Transmitters weighed 2.1 g (lifespan ± 10 weeks) which followed welfare recommendations not to exceed 3% of body weight of tagged individuals (e.g., [63]); in practice, transmitters averaged 2% of male body weight. At least five days were left after marked birds had been released before telemetry recordings were made towards assessment of hunting range so that data recorded should not be influenced by a direct effect of tagging [4,7,63]. Polygyny in two individuals was detected by trapping each of them at two different nestboxes, and later also by radio-tracking when they visited both nests.

We radio-tracked each male for an average of 4.7 ± 1.7 nights (± SD; range 1–8 nights) and for the complete night-time period, i.e. from dusk till dawn. Within tracking nights, two observers (MK and VT) continuously followed each male, recording locations/fixes every 10 minutes (if possible). Observers were connected via walkie-talkies recording exact time of every single fix, their own positions, direction to the tag/male using a compass and the strength of the signal received by using MVT-9000 receivers (Yupiteru Industries Co. Ltd., Japan) and 3-element Yagi antennas. Afterwards, each individual location was confirmed by triangulation in ArcGIS 9.3 software. Experimental calibrations in the field suggested that location accuracy was approximately 100 m (fixes where we were not sure about their sufficient precision were discarded from the analysis).

Home range size was estimated by 80%, 95%, and 100% minimum convex polygon method (MCP; [54,64]) and by kernel density estimator (KDE; [58,65]) with fixed smoothing parameter *h* established by least squares cross-validation method (LSCV; [5,66,67]); HRs were calculated for both 90% and 95% isopleth [67]. The HR sizes were not dependent neither on the number of locations used for their calculation, number of radio-tracking nights nor on the duration of whole radio-tracking period for individual males (number of days between the first and the last night of radio-tacking) in either of the models subsequently used in analysis (GLMM I and II; see Result section below). For this reason, we decided not to exclude the four males/HRs with lowest number of locations (20, 22, 25 and 35) from analyses; HRs for other males were based on more than 58 locations. Since our data sets ranged from 20 to 167 locations per male (see Table 1) we followed the approach of Santangeli et al. [34] and calculated separately a LSCV smoothing parameter for each individual male (range 51.5–149.7), and then took the median of these values (median = 110.4). The median value obtained was used as the smoothing parameter to estimate the HRs which were than comparable among individuals [7,34]. Both types of HR estimates (MCP and KDE) were calculated in Home Range Tools and Hawth’s Tools [68,69] which are freeware extensions for ArcGIS 9.x software. After De Solla et al. [70] and others (e.g., [67,71]), we used fixed time interval of recording to maximize the number of observations included in HR estimations; for our purposes in estimating HRs, locational fixes did not require serial independence of observations [72].

**Table 1:** Home ranges of Tengmalm’s owl males.

	range	mean ± SD	range	mean ± SD	range	mean ± SD	range	mean ± SD	range	mean ± SD
Year	2006		2007^1^		2008		2009		2010	
No. of radio-tracked males	n = 5 individuals		n = 4 individuals		n = 4 individuals		n = 2 individuals		n = 5 individuals	
Date of radio-tracking	19.5.–29.6.		3.5.–27.6.		18.5.–28.6.		1.–16.6.		30.5.–24.7.	
Duration of tracking period (days)	7–26	14.0 ± 6.5	3–20	10.3 ± 6.2	6–16	11.0 ± 3.8	5–6	5.5 ± 0.5	2–23	9.6 ± 7.9
No. of radio-tracking nights	4–7	5.4 ± 1.0	2–8	4.3 ± 2.5	5	5.0 ± 0.0	4–5	4.5 ± 0.5	1–6	4.2 ± 2.2
No. of locations/fixes	76–167	126 ± 29	20–138	65 ± 45	107–149	129 ± 15	59–90	75 ± 16	22–135	87 ± 52
KDE 90% (ha)	107.0–205.5	150.5 ± 35.2	109.7–207.3	162.9 ± 34.9	153.0–247.6	212.0 ± 35.7	129.4–150.3	139.9 ± 10.5	63.7–216.4	108.9 ± 58.2
KDE 95% (ha)	129.9–263.4	189.0 ± 47.8	140.8–263.7	206.0 ± 43.7	181.6–303.3	256.4 ± 45.6	159.3–181.8	170.6 ± 11.2	79.1–265.1	135.5 ± 69.8
MCP 80% (ha)	71.5–132.2	90.2 ± 24.3	30.7–186.4	95.0 ± 56.8	89.7–214.3	157.1 ± 45.5	81.2–88.6	84.9 ± 3.7	20.8–119.6	51.1 ± 37.9
MCP 95% (ha)	83.2–229.6	152.8 ± 58.0	114.9–271.2	176.4 ± 60.4	129.2–294.9	225.4 ± 60.4	117.4–120.0	118.7 ± 1.3	24.7–242.1	86.6 ± 82.2
MCP 100% (ha)	86.8–304.5	190.7 ± 79.0	128.3–304.6	206.4 ± 66.6	147.1–343.2	250.2 ± 69.9	133.1–139.3	136.2 ± 3.1	35.1–251.4	107.0 ± 78.4
Date of nesting (± days)	13.4.–12.5.	23.4. ± 11	12.3.–16.5.	1.4. ± 23	20.3.–12.4.	29.3. ± 9	8.–15.4.	12.4. ± 4	30.3.–31.5.	2.5. ± 27
Date of hatching (± days)	11.5.–10.6.	21.5. ± 11	9.4.–13.6.	29.4. ± 23	20.4.–12.5.	29.4. ± 9	6.–13.5.	10.5. ± 4	28.4.–28.6.	31.5. ± 27
No. of eggs	4–6	4.8 ± 0.7	3–7	5.2 ± 1.3	2–5	3.8 ± 1.1	3–4	3.5 ± 0.5	5–8	6.4 ± 1.2
No. of hatchlings	3–6	4.4 ± 1.0	2–7	4.7 ± 1.6	2–5	3.5 ± 1.1	3–4	3.5 ± 0.5	5–8	6.2 ± 1.2
No. of fledglings	0–3	1.6 ± 1.2	2–7	4.2 ± 1.5	2–5	3.5 ± 1.1	0–1	0.5 ± 0.5	0–7	4.4 ± 2.7
Polygamy (no: yes)	5: 0		2: 2^2^		4: 0		2: 0		5: 0	
Male's age (years; 1: 2: 3+)	1: 4: 0		0: 0: 4		1: 0: 3		1: 0: 1		0: 1: 2^3^	
Male's wing length (mm)	165–174	169 ± 3	164–175	168 ± 4	158–168	164 ± 4	167–169	168 ± 1	161–170	165 ± 3
Male's weight (g)	103–114	108 ± 4	110–130	116 ± 8	96–106	101 ± 4	102–106	104 ± 2	97–109	103 ± 4
Mean age of offspring (days)	12–31	20 ± 6	11–37	29 ± 11	5–43	27 ± 14	28–31	29 ± 2	17–41	28 ± 9
Mean daily precipitation (mm)	0.5–6.8	3.4 ± 2.3	0.0–1.2	0.7 ± 0.4	0.2–3.8	1.7 ± 1.4	1.0–7.6	4.3 ± 3.3	0.1–17.0	4.5 ± 6.3
Mean wind speed (m/s)	2.6–7.2	4.0 ± 1.8	3.0–3.9	3.4 ± 0.4	2.2–4.2	3.2 ± 0.8	5.6–5.9	5.7 ± 0.1	2.2–5.7	3.4 ± 1.4
Mean daily temperature (°C)	9.4–18.3	14.3 ± 3.5	12.7–16.6	15.1 ± 1.5	11.4–15.6	13.9 ± 1.7	8.4–11.7	10.0 ± 1.7	9.3–24.8	17.0 ± 5.1
Prey abundance	0.28		6.52		1.19		1.10		10.19	

Home range sizes estimated by 90% and 95% fixed kernel density estimator and 80%, 95%, and 100% minimum convex polygon method, and the list of fixed effects used in the GLMM I and II for the hunting home range size of male Tengmalm’s owls during the breeding season.

^1^ Data from 2007 regards four individual males but six individual nests because two males were polygynous.

^2^ Between nestbox distances belonging to the two polygynous males were 410 and 1035 meters.

^3^ In 2010 only three males were aged.

Prey abundance (small mammals) in the study area was assessed by using snap-traps at the beginning of June during all study years; snap-traps were set up in three 1 ha squares (with 10 m spacing). The traps were left out for 3 nights and checked daily in the morning. The total trapping effort was 1089 trap nights (n = 3 locations). The number of mammals captured per 100 traps-nights was calculated as an index of prey abundance. All trapped individuals (n = 193 in total; 3, 71, 13, 12 and 94 in 2006–2010, respectively) were identified to the species level. For details of prey abundance in different study years see Table 1.

Owls were trapped, handled and tagged under permit No. 530/758 R/08-Abt/UL from the Ministry of the Environment of the Czech Republic, and were ringed under the Ringing Centre of the National Museum in Prague permit No. 329; all efforts were made to minimize suffering.

### Statistical analyses

All data were analysed with the aid of SAS System version 9.4 (SAS Institute Inc.). The analysis was made in two steps. In order to check for possible multicollinearity we first calculated correlations between the individual variables involved (listed in Table 1). Significant correlation was found between the date of nesting/hatching and prey abundance (0.69, P = 0.0003), between the number of eggs (E), hatchlings (H) and fledglings (F)–(EH: 0.91, P<0.0001; EF: 0.59, P = 0.0036; HF: 0.57, P = 0.0056), mean wind speed and daily precipitation (0.54, P = 0.0083), and mean wind speed and mean daily temperature (-0.73, P<0.0001). We subsequently made a judgment of the extent of intercorrelation and collinearity by checking related statistics, such as tolerance value or variance inflation factor (VIF), Eigenvalue, and condition number following the approach of Belsley et al. [73] and using TOL, VIF and COLLIN options of the MODEL statement in the SAS REG procedure. Low eigenvalues and large condition indices indicated that date of nesting and hatching, number of eggs and hatchlings, and mean daily temperature and daily precipitation, were redundant and therefore we omitted these variables from later analyses.

Associations were subsequently sought between individual male hunting HR size during breeding season and the remaining variables (fixed and random effects, see below) using a multivariate General Linear Mixed Model (GLMM, PROC MIXED, SAS, version 9.4). To account for the use of repeated measures on the same individuals, all analyses were performed using mixed model analysis with individual male as a random factor. We constructed the GLMM entering first the factor and/or factors expected to have the most significant effect, subsequently checking the model with addition of other factors which might contribute. The significance of each fixed effect in the mixed GLMM was assessed by the F-test. Non-significant factors (P > 0.05) were dropped from the model. Where appropriate we tested interaction terms. Associations between the dependent variable and fixed effects were estimated by fitting a random coefficient model using PROC MIXED as described by Tao et al. [74]. We calculated predicted values of the dependent variable and plotted them against the fixed effects with predicted regression lines.

In the analyses the size of individual male hunting HRs during breeding season established by (I) KDE and (II) MCP method were taken as the dependent variable. The following factors were considered as fixed effects in both models: number of fledglings, mean age of nestlings/fledgling from hatching (days), presence or absence of polygyny, age of male (1, 2 or ≥3 years), number of radio-tracking nights, number of locations/fixes used for HR calculation, duration of whole radio-tracking period for individual males (days), male’s weight (g) and wing length (mm), mean wind speed (m/s) and prey abundance (see Table 1). All relevant data used in the analyses (GLMM I and II) are presented in S1 Table.

Dependent variables and fixed effects entered into both models were log-transformed in order to achieve normal distribution of residuals. Interactions tested (prey abundance with mean wind speed and prey abundance with number of fledged individuals) were not significant, and thus excluded from both models (I and II).

## Results

The mean size of hunting HRs during breeding season for Tengmalm’s owl males (n = 20) calculated according to 90% kernel density estimator was 153.8 ± 53.7 ha (± SD) with a median value of 152.9 ha, and according to 95% KDE: 190.7 ± 65.7 ha with median 187.1 ha; MCP method offered a range estimate of 94.2 ± 53.3 ha (80% MCP) with median 83.1 ha, 152.1 ± 79.8 ha (95% MCP) with median 131.8 ha, and according to 100% MCP: 179.4 ± 87.4 ha with median 156.7 ha. These ranges were based on 99 ± 45 (± SD) locations/fixes on average with median value 121 locations.

**Fig 1:**
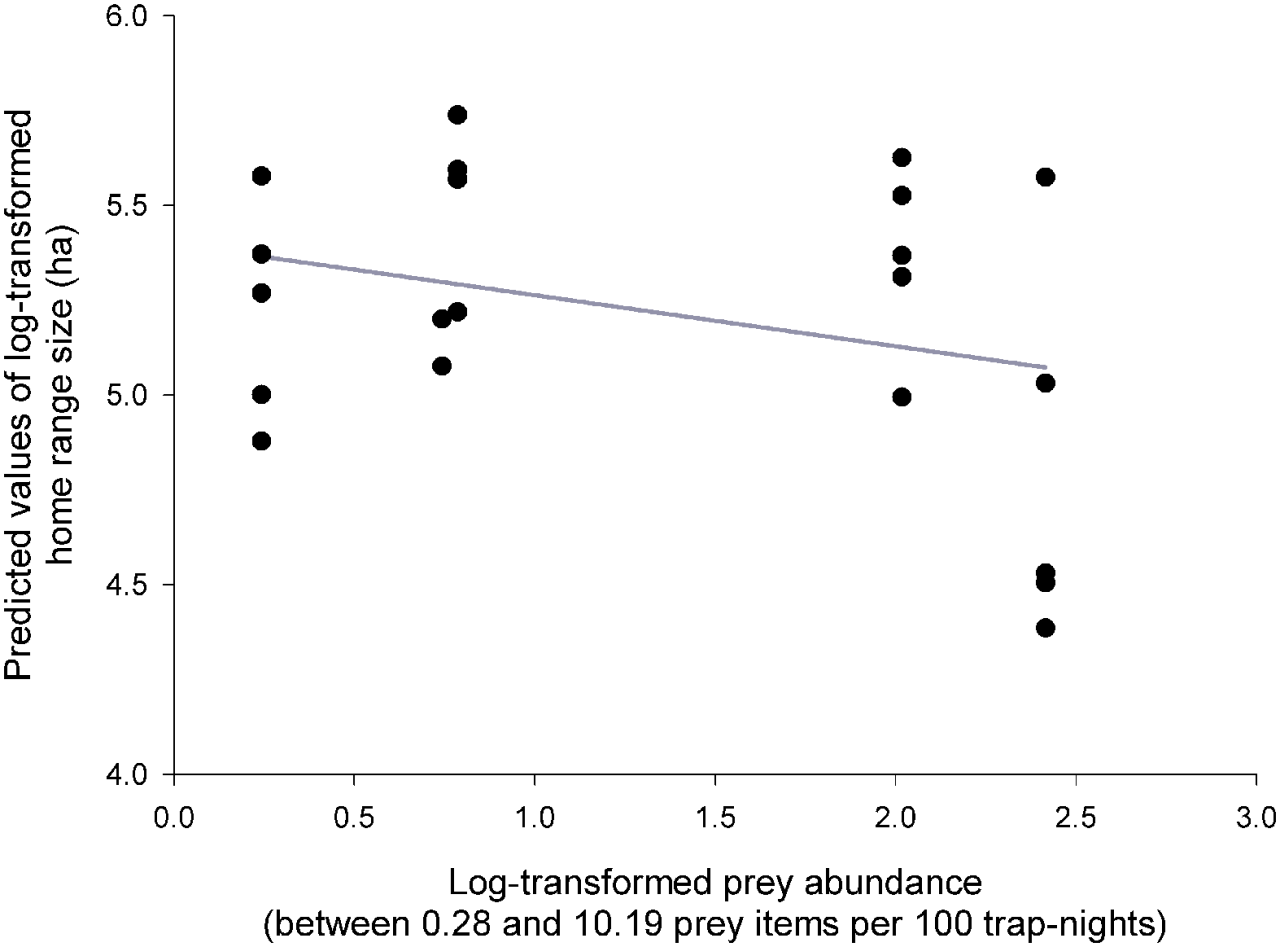
Prey abundance. Predicted values of the size (log-transformed) of male Tengmalm’s owls’ hunting home range during the breeding season established by the 95% fixed kernel density estimator, plotted against an index of prey abundance.

**Fig 2:**
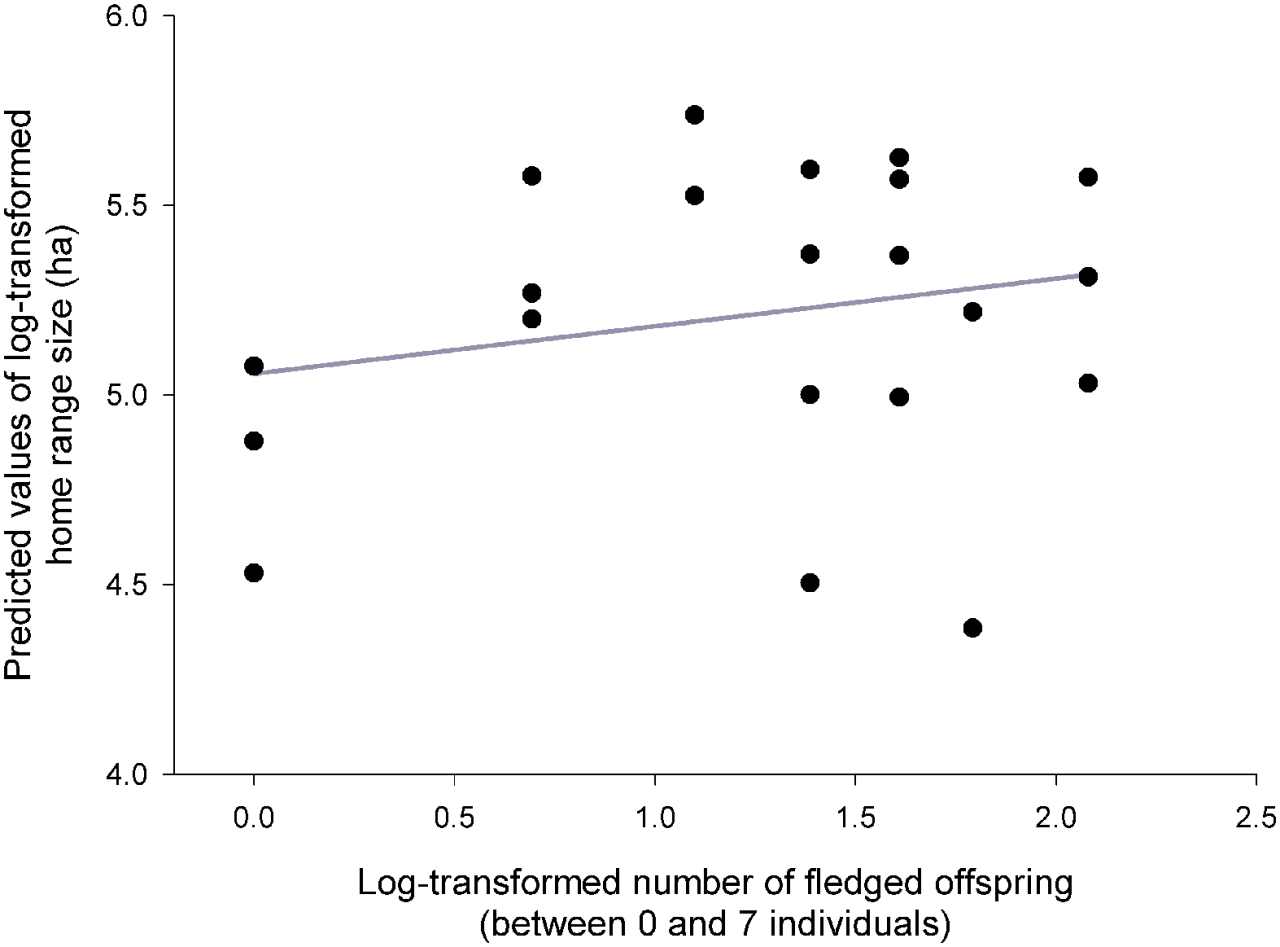
Number of fledglings. Predicted values of the size (log-transformed) of male Tengmalm’s owls’ hunting home range during the breeding season established by the 95% fixed kernel density estimator, plotted against the log-transformed number of fledglings raised.

**Fig 3:**
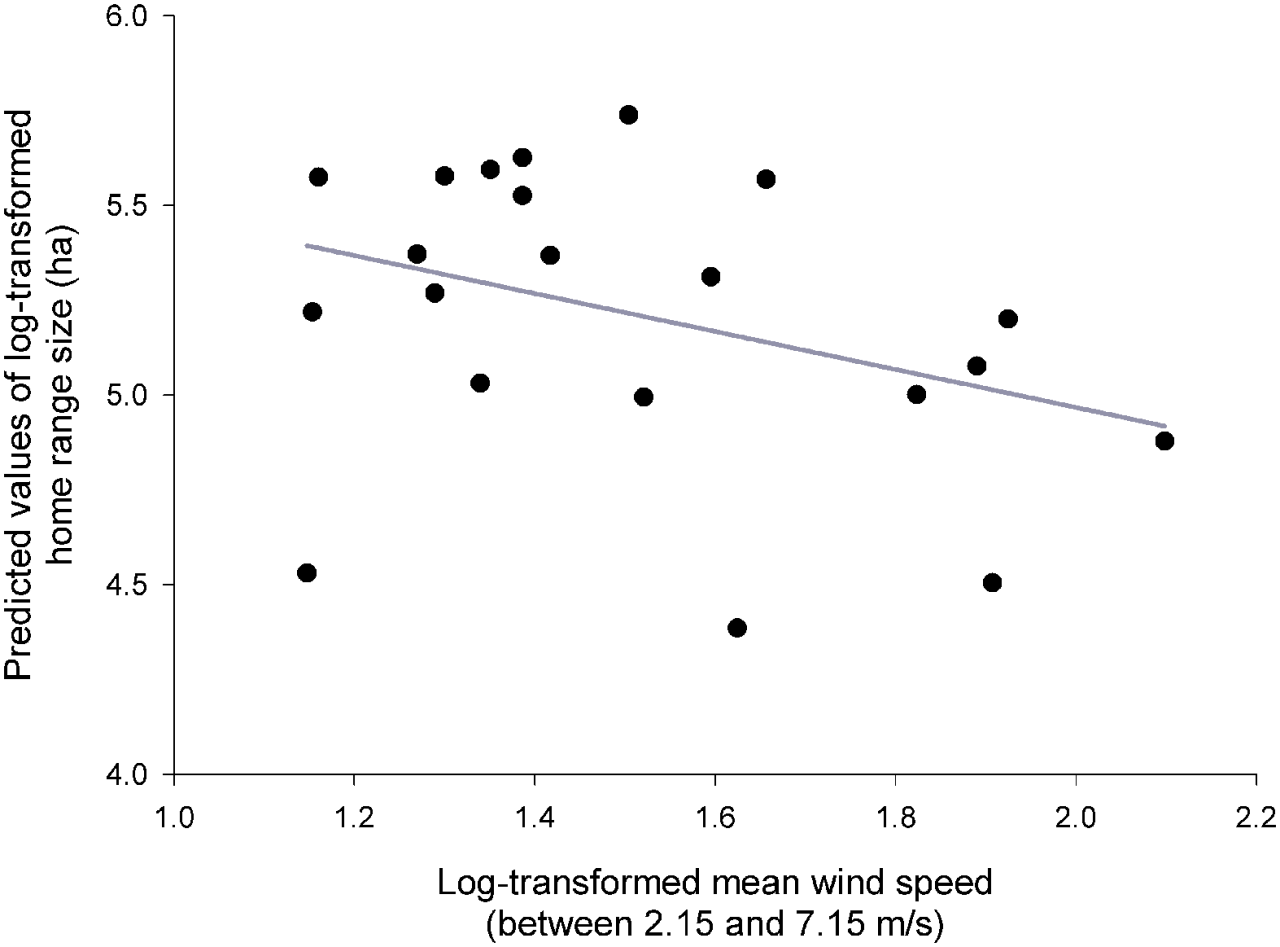
Wind speed. Predicted values of the size (log-transformed) of male Tengmalm’s owls’ hunting home range during the breeding season established by the 95% fixed kernel density estimator, plotted against the mean wind speed (log-transformed values) during particular radio-tracking nights.

Results of the GLMM I (Table 2) revealed that the size of hunting HRs during breeding season established by 95% KDE was dependent on prey abundance (Fig 1), presence or absence of polygyny, number of fledged individuals (Fig 2), and mean wind speed during particular radio-tracking nights (Fig 3). Results were identical for 90% KDE, and therefore are not shown.

**Table 2:** The results of the GLMM I.

Fixed effect–GLMM I	Num DF	Den DF	F value	P =
Log-transformed prey abundance	1	7.81	213.36	0.0001
Presence or absence of polygyny	1	9.74	132.15	0.0001
Log-transformed number of fledglings	1	11.1	9.59	0.0101
Log-transformed mean wind speed	1	10.7	7.82	0.0178

The results of the GLMM I for factors affecting the Tengmalm’s owl males’ hunting home range size during the breeding season established by the 95% fixed kernel density estimator.

Home range sizes increased with decreasing prey abundance (Fig 1). Polygynously mated males had significantly larger HRs in comparison with males mated monogamously. Size of hunting range also increased significantly with increasing number of successfully fledged offspring (Fig 2), and decreased with increasing mean wind speed (Fig 3).

Results of the GLMM II (where size of hunting range was defined from 95% or 100% MCP) were virtually identical regarding every single fixed effect (Table 2); graphical representations of relationships were also very similar to figures presented in Figs 1–3, and are thus not repeated here.

## Discussion

The size of hunting HRs of male Tengmalm’s owls during the breeding season reported in this study is consistent with results from previous studies by other authors [34,55–57] who reported range sizes from 100–300 ha. Compared to these studies, our owls occupied a rather unusual mountain habitat that had in the past been severely damaged by air-pollution and now consisted of a mosaic of open areas, fragments of young secondary non-native blue spruce stands, and small patches of tall old-growth Norway spruce. Thus, studies from various parts of Europe have shown that the sizes of hunting HRs are very similar despite marked variation in habitat and/or different methods used for HR calculations, and it seems there is no fundamental difference in hunting HR size in Tengmalm’s owl during breeding at the population level (populations of Scandinavia, Central and Western Europe). However, it should be noted that we followed males for several nights only (4.7 nights on average) as it was done also in above mentioned studies. It is possible that tracked individuals did not visit every part of their HR during relatively short tracking period, and thus, our and their results could be a mixture of HR sizes and HR used patterns.

We found the HR size was dependent on different prey abundance as expected (prediction i). This is in accordance with other studies on birds of prey which reported larger HRs during poor food years and vice versa [35,36,75], and this is the first study documenting such effect in Tengmalm’s owl males during breeding period.

We have reported a similar relationship also for Tengmalm’s owl fledglings during the PFDP in the same study area that in the season with low prey abundance young owls occupied larger HRs than in the year with higher prey abundance [49]. However, compared to our present results, Santangeli et al. [34] did not find that food supplementation affected hunting HR size in Tengmalm’s owl males during breeding phase. In their study, HR size was affected by habitat structure and decreased with cover of spruce forest, which is denser in structure and richer in prey than pine forest and especially clear-cut areas [34]. These contradictory findings might thus be due to the differences in habitat structure between both study areas. Another explanation could be that Santangeli et al. [34] radio-tracked males during the beginning of the PFDP but supplementary food was offered only until fledging. This fact could confuse the effect of food supplementation on HR size between food supplemented and control nests because all males already hunted to the full extent at the beginning of the PFDP.

We also found the HR size was dependent on presence or absence of polygyny (prediction ii). Although data for only two polygynous males were available, these males have significantly larger HRs compared to those mated monogamously most as a result of having to move between their two nestboxes (being 410 and 1035 m apart). Large size of HRs of these two polygynous males could further be exaggerated by the fact that they were supporting a large number of offspring, since hunting HR size was also positively associated with the number of fledged individuals (prediction iii).

No such relationship was found in the ferruginous hawk [76], while Pfeiffer and Meyburg [36] reported a significant negative correlation between HR size and number of young fledged for the red kite. It would thus appear that both options are possible. We suggest that the contradictory findings could be explained by partly distinct diet habits, the number of young which these species commonly care for, and the overall size of hunting HR used. The Tengmalm’s owl feeds primarily on small rodents, lays six eggs on average with a mean hunting HR during breeding season covering ca. 2 km^2^ ([34,43,44], this study). The red kite which takes a wide range of different foods, lays two eggs on average with a mean hunting HR during breeding season covering ca. 64 km^2^ [36,77]. One must assume that the negative correlation between HR size and brood size reported is a consequence of the fact that when food is plentiful the birds do not have to hunt over such large areas and have sufficient resources also to raise a larger number of young; since they search for food over a relatively large area, we suggest that the situation is quite distinct from that facing the Tengmalm’s owl.

We did not find support for the fourth prediction (iv) that the HR size should vary positively with the actual age of nestlings/fledglings, despite the fact that Tengmalm’s owl males increased feeding rates throughout the nestling period [78]. The reason might be that the differences in offspring age between particular nests were simply not large enough (26 ± 10 days from hatching; mean ± SD). The HRs in this study were in most cases recorded during the late nestling phase and/or at the very beginning of the PFDP. We suggest the HRs might be seen to differ in size according to this prediction if some of them were recorded during pre-laying and/or incubation period and compared with ranges registered during the nestling phase and/or PFDP as we have done here. However, no significant differences in HR size were recorded among pre-laying, incubation and early nestling period in peregrine falcon [38], but their HRs tripled in size after the chicks fledged. We speculate that similar enlargement of male hunting HR size and/or greater difference in HR sizes between good and poor food years might be detected in Tengmalm’s owl if the ranges would be recorded throughout or at the end of the PFDP. This would be consistent with different movement patterns of Tengmalm’s owl fledglings who were located more distantly from their nestboxes during poor food year compared to good one [49].

Finally, our results suggested that the HR size decreased with increasing wind speed and/or amount of precipitation during the radio-tracking nights when the HRs were recorded (prediction v). This reflects the hunting strategy of Tengmalm’s owl. The owl searches for prey by the pause-travel mode and depends heavily on sound to localize ground-dwelling prey [51–53]. Thus, high wind speed or heavy rain very likely hamper the hunt itself. This agrees with the observations of Klaus et al. [60] who described that nest feeding visits in Tengmalm’s owl decreased during rain. We also recorded decreasing frequency of begging calls by fledglings with increasing precipitation [79]. Therefore, it could be advantageous for the Tengmalm’s owl to remain on a hunting perch for longer than just for the usual two minutes [52] because under such conditions hunting success depends more on chance, and waiting for prey on one place is at least energy expenditure saving. Moreover, Tengmalm’s owl males are able to apply loose-shift (avoiding of unsuccessful hunting sites between consecutive nights) and win-stay (returning to successful hunting sites within and/or between nights) strategy while hunting [55], and we suggest both strategies should also be of value during windy and/or rainy nights, and increasingly so the longer they stay on every perch.

To conclude: we stress the importance of the time interval during which the HRs are recorded as shown for instance by studies regarding temporal changes in range use within years and/or between different parts of breeding season (e.g., [38,80,81]). We detected that hunting ranges during the breeding season were larger when less prey was available, and further that polygynously mated males, and those with more fledglings had overall larger HRs than males mated monogamously and/or with fewer raised fledglings. We also found that hunting ranges recorded in harsh weather conditions, and high wind speed and/or heavy rain in particular, were smaller than those registered during better weather. Finally, our results provide novel insights into what factors may influence HR size in male Tengmalm’s owls and emphasize the importance of prey abundance as a key factor.

## Supporting information

S1 Table Supporting information. Relevant data used in the analyses (GLMM I and II).
(XLS)

## References

[1] Darwin C (1861) On the origin of species by means of natural selection. London: Murray.

[2] Powell RA (2000) Animal home ranges and territories and home range estimators In: Boitani L, Fuller T, eds. Research techniques in animal ecology: controversies and consequences. New York, USA: Columbia University Press, New York 65–110.

[3] Burt WH (1943) Territoriality and home range concepts as applied to mammals. J Mammal 24: 346–352.

[4] White GC, Garrott RA (1990) Analysis of wildlife radio-tracking data. San Diego: Academic Press.

[5] Seaman DE, Powell RA (1996) An evaluation of the accuracy of kernel density estimators for home range analysis. Ecology 77: 2075–2085.

[6] Hansteen TL, Andreassen HP, Ims RA (1997) Effects of spatiotemporal scale on autocorrelation and home range estimators. J Wildl Manage 61: 280–290.

[7] Kenward RE (2001) A manual for wildlife radio tagging. London: Academic Press.

[8] Harris S, Cresswell WJ, Forde PG, Trewhella WJ, Woollard T, Wray S (1990) Home-range analysis using radio-tracking data: A review of problems and techniques particularly as applied to the study of mammals. Mammal Rev 20: 97–123.

[9] Laver PN, Kelly MJ (2008) A critical review of home range studies. J Wildl Manage 72: 290–298.

[10] McLoughlin PD, Ferguson SH (2000) A hierarchical pattern of limiting factors helps explain variation in home range size. Ecoscience 7: 123–130.

[11] Mace GM, Harvey PH (1983) Energetic constraints on home-range size. Am Nat 121: 120–132.

[12] Adams ES (2001) Approaches to the study of territory size and shape. Annu Rev Ecol Evol Syst 32: 277–303.

[13] Allen TFH, Starr TB (1982) Hierarchy: perspectives for ecological complexity. Chicago, Illinois: University of Chicago Press.

[14] McNab BK (1963) Bioenergetics and determination of home range size. Am Nat 97: 133–140.

[15] Schoener TW (1968) Sizes of feeding territories among birds. Ecology 49: 123–141.

[16] Turner FB, Jennrich RI, Weintraub JD (1969) Home ranges and body size of lizards. Ecology 50: 1076–1081.

[17] Mace GM, Harvey PH, Clutton-Brock TH (1984) Vertebrate home range size and energetic requirements In: Swingland IR, Greenwood PJ, eds. The ecology of animal movement. Oxford: Clarendon Press 32–53.

[18] Hixon MA (1980) Food-production and competitor density as the determinants of feeding territory size. Am Nat 115: 510–530.

[19] Taitt MJ, Krebs CJ (1982) Manipulation of female behavior in field populations of *Microtus townsendii*. J Anim Ecol 51: 681–690.

[20] Tufto J, Andersen R, Linnell JDC (1996) Habitat use and ecological correlates of home range size in a small cervid: The Roe Deer. J Anim Ecol 65: 715–724.

[21] Powell RA, Zimmerman JW, Seaman DE (1997) Ecology and behaviour of North American Black Bears. New York: Chapman and Hall.

[22] Macdonald DW (1983) The ecology of carnivore social behaviour. Nature 301: 379–384.

[23] Kruuk H, Parish T (1982) Factors affecting population density, group size and territory size of the European badger, *Meles meles*. J Zool 196: 31–39.

[24] Ross S, Munkhtsog B, Harris S (2012) Determinants of mesocarnivore range use: relative effects of prey and habitat properties on Pallas's cat home range size. J Mammal 93: 1292–1300.

[25] Dahle B, Støen OG, Swenson JE (2006) Factors influencing home range size in subadult brown bears. J Mammal 87: 859–865.

[26] Anderson DP, Forester JD, Turner MG, Frair JL, Merrill EH, Fortin D, et al (2005) Factors influencing female home range sizes in elk (*Cervus elaphus*) in North American landscapes. Landsc Ecol 20: 257–271.

[27] Jerina K (2012) Roads and supplemental feeding affect home range size of Slovenian red deer more than natural factors. J Mammal 93: 1139–1148.

[28] Scillitani L, Sturaro E, Monaco A, Rossi L, Ramanzin M (2012) Factors affecting home range size of male Alpine ibex (*Capra ibex ibex*) in the Marmolada massif. Hystrix 23: 19–27.

[29] Hubbs AH, Boonstra R (1998) Effects of food and predators on the home range sizes of Arctic ground squirrels (*Spermophilus parryii*). Can J Zool 76: 592–596.

[30] Breininger DR, Bolt MR, Legare ML, Drese JH, Stolen ED (2011) Factors influencing home range sizes of eastern indigo snakes in Central Florida. J Herpetol 45: 484–490.

[31] Belthoff JR, Sparks EJ, Ritchison G (1993) Home ranges of adult and juvenile Eastern Screech-Owls: size, seasonal variation and extent of overlap. J Raptor Res 27: 8–15.

[32] Grzywaczewski G (2009) Home range size and habitat use of the Little Owl *Athene noctua* in East Poland. Ardea 97: 541–545.

[33] Schilling JW, Dugger KM, Anthony RG (2013) Survival and home-range size of Northern Spotted Owls in southwestern Oregon. J Raptor Res 47: 1–14.

[34] Santangeli A, Hakkarainen H, Laaksonen T, Korpimäki E (2012) Home range size is determined by habitat composition but feeding rate by food availability in male Tengmalm's Owls. Anim Behav 83: 1115–1123.

[35] Zabel CJ, McKelvey K, Ward JP (1995) Influence of primary prey on home-range size and habitat-use patterns of Northern Spotted Owls (*Strix occidentalis caurina*). Can J Zool-Rev Can Zool 73: 433–439.

[36] Pfeiffer T, Meyburg BU (2015) GPS tracking of Red Kites (*Milvus milvus*) reveals fledgling number is negatively correlated with home range size. J Ornithol 156: 963–975.

[37] Forsman ED, Kaminski TJ, Lewis JC, Maurice KJ, Sovern SG, Ferland C, et al (2005) Home range and habitat use of Northern Spotted Owls on the Olympic Peninsula, Washington. J Raptor Res 39: 365–377.

[38] Sokolov V, Lecomte N, Sokolov A, Rahman ML, Dixon A (2014) Site fidelity and home range variation during the breeding season of Peregrine Falcons (*Falco peregrinus*) in Yamal, Russia. Polar Biol 37: 1621–1631.

[39] Redpath SM (1995) Habitat fragmentation and the individual—Tawny Owls *Strix aluco* in woodland patches. J Anim Ecol 64: 652–661.

[40] Hinam HL, Clair CCS (2008) High levels of habitat loss and fragmentation limit reproductive success by reducing home range size and provisioning rates of Northern Saw-Whet Owls. Biol Conserv 141: 524–535.

[41] Cramp S (1985) The birds of the western Palaearctic, Vol. IV Oxford: Oxford University Press.

[42] Korpimäki E (1981) On the ecology and biology of Tengmalm's Owl (*Aegolius funereus*) in southern Ostrobothnia and Soumenselkä, western Finland: Acta Univ Oul A 118 Biol 13: 1–84.

[43] Korpimäki E, Hakkarainen H (2012) The Boreal Owl: ecology, behaviour and conservation of a forest-dwelling predator. Cambridge: Cambridge University Press.

[44] König C, Weick F (2008) Owls of the world. Second edition New Haven and London: Yale University Press.

[45] Kouba M, Bartoš L, Korpimäki E, Zárybnická M (2015) Factors affecting the duration of nestling period and fledging order in Tengmalm's Owl (*Aegolius funereus*): Effect of wing length and hatching sequence. PLoS One 10(3): e0121641 10.1371/journal.pone.0121641 25793880 PMC4368509

[46] Eldegard K, Sonerud GA (2009) Female offspring desertion and male-only care increase with natural and experimental increase in food abundance. Proc R Soc B-Biol Sci 276: 1713–1721. 10.1098/rspb.2008.1775 PMC2660990 19324835

[47] Eldegard K, Sonerud GA (2010) Experimental increase in food supply influences the outcome of within-family conflicts in Tengmalm's Owl. Behav Ecol Sociobiol 64: 815–826.

[48] Eldegard K, Sonerud GA (2012) Sex roles during post-fledging care in birds: female Tengmalm's Owls contribute little to food provisioning. J Ornithol 153: 385–398.

[49] Kouba M, Bartoš L, Šťastný K (2013) Differential movement patterns of juvenile Tengmalm's Owls (*Aegolius funereus*) during the post-fledging dependence period in two years with contrasting prey abundance. PLoS One 8(7): e67034 10.1371/journal.pone.0067034 23843981 PMC3700927

[50] Zárybnická M (2009) Parental investment of female Tengmalm's Owls *Aegolius funereus*: correlation with varying food abundance and reproductive success. Acta Ornithol 44: 81–88.

[51] Norberg RA (1970) Hunting technique of Tengmalm’s Owl *Aegolius funereus*. Ornis Scand 1: 51–64.

[52] Bye FN, Jacobsen BV, Sonerud GA (1992) Auditory prey location in a pause-travel predator—search height, search time, and attack range of Tengmalm's Owls (*Aegolius funereus*). Behav Ecol 3: 266–276.

[53] Andersson M (1981) On optimal predator search. Theor Popul Biol 19: 58–86.

[54] Mohr CO (1947) Table of equivalent populations of north american small mammals. Am Midl Nat 37: 223–249.

[55] Sonerud GA, Solheim R, Jacobsen BV (1986) Home-range use and habitat selection during hunting in a male Tengmalm's Owl *Aegolius funereus*. Fauna norv Ser C, Cinclus 9: 100–106.

[56] Jacobsen BV, Sonerud GA (1987) Home range of Tengmalm´s Owl: A comparison between nocturnal hunting and diurnal roosting. USDA For Serv Gen Tech Rep RM 142: 189–192.

[57] Sorbi S (2003) Size and use of Tengmalm's Owl *Aegolius funereus* home range in the high Belgian Ardennes: Results from radio-tracking (In French with English summary). Alauda 71: 215–220.

[58] Silverman BW (1986) Density estimation for statistics and data analysis. London: Chapman and Hall.

[59] Hakkarainen H, Mykra S, Kurki S, Korpimäki E, Nikula A, Koivunen V (2003) Habitat composition as a determinant of reproductive success of Tengmalm's Owls under fluctuating food conditions. Oikos 100: 162–171.

[60] Klaus S, Mikkola H, Wiesner J (1975) Aktivität und Ernährung des Rauhfusskauzes *Aegolius funeres* (L.) während der Fortpflanzungsperiode. Zool Jb Syst Bd 102: 485–507.

[61] Zárybnická M, Riegert J, Sťastný K (2015) Non-native spruce plantations represent a suitable habitat for Tengmalm's Owl (*Aegolius funereus*) in the Czech Republic, Central Europe. J Ornithol 156: 457–468.

[62] Hörnfeldt B, Carlsson BG, Nordstrom A (1988) Molt of primaries and age-determination in Tengmalm's Owl (*Aegolius funereus*). Auk 105: 783–789.

[63] Withey JC, Bloxton TD, Marzluff JM (2001) Effects of tagging and location error in wildlife radiotelemetry studies In: Millspaugh JJ, Marzluff JM, eds. Radio tracking and animal populations. San Diego: Academic Press 43–70.

[64] Hayne DW (1949) Calculation of size of home range. J Mammal 30: 1–18.

[65] Worton BJ (1989) Kernel methods for estimating the utilization distribution in home-range studies. Ecology 70: 164–168.

[66] Seaman DE, Millspaugh JJ, Kernohan BJ, Brundige GC, Raedeke KJ, Gitzen RA (1999) Effects of sample size on kernel home range estimates. J Wildl Manage 63: 739–747.

[67] Börger L, Franconi N, De Michele G, Gantz A, Meschi F, Manica A, et al (2006) Effects of sampling regime on the mean and variance of home range size estimates. J Anim Ecol 75: 1393–1405. 10.1111/j.1365-2656.2006.01164.x 17032372

[68] Rodgers AR, Carr AP, Beyer HL, Smith L, Kie JG (2007) HRT: Home Range Tools for ArcGIS. Version 1.1 Thunder Bay, Ontario, Canada: Ontario Ministry of Natural Resources, Centre for Northern Forest Ecosystem Research.

[69] Rodgers AR, Kie JG (2011) HRT: Home Range tools for ArcGIS, A User’s Manual. Ontario: Centre for Northern Forest Ecosystem Research.

[70] De Solla SR, Bonduriansky R, Brooks RJ (1999) Eliminating autocorrelation reduces biological relevance of home range estimates. J Anim Ecol 68: 221–234.

[71] Cushman SA, Chase M, Griffin C (2005) Elephants in space and time. Oikos 109: 331–341.

[72] Hurlbert SH (1984) Pseudoreplication and the design of ecological field experiments. Ecol Monogr 54: 187–211.

[73] Belsley DA, Kuh E, Welsch RE (1980) Regression diagnostics: Identifying influential data and sources of collinearity. New York: John Wiley & Sons.

[74] Tao J, Littel R, Patetta M, Truxillo C, Wolfinger R (2002) Mixed model analyses using the SAS system course notes. Carry, NC, USA: SAS Institute Inc.

[75] Newton I (1986) The Sparrowhawk. Calton: Poyser.

[76] Leary AW, Mazaika R, Bechard MJ (1998) Factors affecting the size of Ferruginous Hawk home ranges. Wilson Bull 110: 198–205.

[77] Ferguson-Lees J, Christie DA (2001) Raptors of the world. London: Christopher Helm.

[78] Zárybnická M, Korpimäki E, Griesser M (2012) Dark or short nights: differential latitudinal constraints in nestling provisioning patterns of a nocturnally hunting bird species. PLoS One 7(5): e36932 10.1371/journal.pone.0036932 22615850 PMC3353992

[79] Kouba M, Bartoš L, Šťastný K (2014) Factors affecting vocalization in Tengmalm's Owl (*Aegolius funereus*) fledglings during post-fledging dependence period: scramble competition or honest signalling of need? PLoS One 9(4): e95594 10.1371/journal.pone.0095594 24760102 PMC3997414

[80] Rivers JW, Johnson JM, Haig SM, Schwarz CJ, Burnett LJ, Brandt J, et al (2014) An analysis of monthly home range size in the critically endangered California Condor *Gymnogyps californianus*. Bird Conserv Int 24: 492–504.

[81] Wiktander U, Olsson O, Nilsson SG (2001) Seasonal variation in home-range size, and habitat area requirement of the Lesser Spotted Woodpecker (*Dendrocopos minor*) in southern Sweden. Biol Conserv 100: 387–395.

